# Confidence, animal spirits, and the macroeconomy in China: Based on mixed-frequency data models

**DOI:** 10.1371/journal.pone.0332909

**Published:** 2025-09-19

**Authors:** Wanbo Lu, Qibo Liu, Haofang Li

**Affiliations:** 1 School of Management Science and Engineering, Southwestern University of Finance and Economics, Chengdu, Sichuan, China; 2 School of Statistics and Data Science, Southwestern University of Finance and Economics, Chengdu, Sichuan, China,; 3 Beijing Zitiao Network Technology Co., Ltd., Beijing, China; Alexandru Ioan Cuza University: Universitatea Alexandru Ioan Cuza din Iasi, ROMANIA

## Abstract

This paper employs the mixed-frequency Granger causality test, reverse unconstrained mixed-frequency data sampling models, and Chinese data from January 2006 to June 2024 to test the nexus between consumer confidence and the macroeconomy. The results show that changes in the real estate market, GDP, and urban unemployment rate are Granger causes of consumer confidence. In reverse, consumer confidence is a Granger cause of the CPI. Second, GDP and the real estate market (CPI and urban unemployment rate) have a significant positive (negative) impact on consumer confidence, while the conditions of industrial production, interest rate, and stock market do not. Third, the “animal spirits” extracted from consumer confidence cannot lead to noticeable fluctuations in China’s macroeconomy. This suggests that the “animal spirits” will not dominate economic growth, even though they affect the macroeconomy slightly and inevitably. The results are robust after replacing the dependent variable and considering the influence of the global financial crisis and the COVID-19 pandemic.

## 1. Introduction

**Fig 1:**
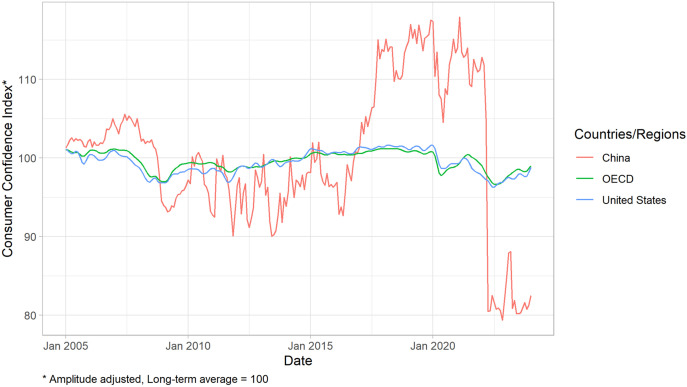
Consumer confidence index time series in the Organization for Economic Co-operation and Development, the United States, and China. The X-axis indicates the date, and the Y-axis indicates the level of the consumer confidence index.

In recent years, affected by COVID-19 pandemic, regional conflicts and the Sino-US trade disputes, the consumer confidence index in the world has been visibly declining, especially for China and the United States (Fig 1). And meanwhile, several studies have explored the impact of confidence on some macroeconomic areas, such as the securities market (Bansal and Shaliastovich [1], Rojo-Suarez and Belen [2], Sekizawa and Konishi [3], Bertella et al. [4]), monetary and fiscal policy (Wang et al. [5], Zhang and Sun [6]), and growth and fluctuation (Taylor and McNabb [7], Bafumi [8], Zhuang et al. [9], Guo and He [10], Zhu and Deng [11]). However, these studies did not reach a consensus on the relationship between confidence and the macroeconomy. Some studies propose that confidence has a significant impact on the macroeconomy (Zhuang et al. [9], Zhu and Deng [11], and Dees and Zimic [12]), while some studies show different results (Chen and Tang [13]). In this context, clarifying the relationship between market psychological factors and the macroeconomy is of great theoretical and practical significance. Specifically, the relationship between confidence and the macroeconomy in China is an important question to answer.

The reason for the inconsistency among the existing studies lies in the measurement limitations, sample size, and econometric model. Specifically, most early studies used small samples or proxy indicators due to the abstraction of the confidence concept and the late start of surveys. This may involve problems with sample size and measurement error. Meanwhile, limited by the econometric model, most existing studies employ the autoregressive distributed lag model (Soric et al. [14]) and the (structural) vector autoregression method (Byrne et al. [15]; Larsen and Thorsrud [16]; Dees and Zimic [12]) to test the relationship between confidence and the macroeconomy. However, all these methods require the data to be sampled at a common frequency (common-frequency data), such as yearly, quarterly, or monthly. A common practice for the data sampled at different frequencies (mixed-frequency data) is temporal aggregation, such as totalling or averaging. However, this temporal aggregation not only reduces the sample size but also may lead to distorted causal relationships between variables (Ghysels et al. [17]). For example, Yuan and Wen [18] found that the difference in the causal relationship between economic growth and confidence is statistically significant when using common-frequency data and mixed-frequency data. Thus, another interesting question is whether the relationship between confidence and the macroeconomy in China is robust in the mixed-frequency data context.

To answer the two questions above, this study adopts the mixed-frequency autoregression (MF-VAR) and reverse unconstrained mixed data sampling (RU-MIDAS) model, which can model the frequency-mismatch data directly, to test the relationship between confidence and the macroeconomy. Specifically, based on MF-VAR, this paper first uses the newest Chinese data from January 2006 to June 2024 to test the Granger causality between confidence and the macroeconomy. Then, it adopts the RU-MIDAS model to test the effects of the macroeconomy on confidence. Meanwhile, the confidence is decomposed into “fundamental confidence” and “animal spirits”. Finally, based on MF-VAR, this paper tests the effect of “animal spirits” on the macroeconomy.

The results show that changes in the real estate market, GDP, and urban unemployment rate are Granger causes of consumer confidence. In reverse, consumer confidence is a Granger cause of the CPI. Second, GDP and the real estate market (CPI and urban unemployment rate) have a significant positive (negative) impact on consumer confidence, while the conditions of industrial production, interest rate, and stock market do not. Third, the “animal spirits” extracted from consumer confidence cannot lead to noticeable fluctuations in China’s macroeconomy. This suggests that the “animal spirits” will not dominate economic growth, even though they affect the macroeconomy slightly and inevitably. The results are robust after replacing the dependent variable and considering the influence of the global financial crisis and the COVID-19 pandemic.

Compared to existing research, the novelties of this study lie in (1) employing mixed-frequency data models with the newest sample to avoid sample size reduction and potential distortion of causal relationships that may arise from temporal aggregation and (2) With the newest sample, this study provide the newest and robust evidence of the relationship between confidence and the economy, especially in the context of mixed-frequency data and the post-COVID-19 era.

This study is organized as follows. First, based on the literature, we theoretically analyze the relationship between confidence and the macroeconomy. Second, empirical design, including variables, data, and model specification, is explained in Section 3. Third, empirical results, such as the Granger causality test, the decomposition of confidence, and the analysis of the nexus between the “animal spirits” and the macroeconomy, are displayed in Section 4. Last, the conclusion and discussion are conducted in Section 5.

## 2. Literature review

### 2.1. The nexus between confidence and the macroeconomy

The economic studies on the psychological factor can be traced back to Jevons W S [19]. He discussed humans’ pleasure and pain and, thus, the measurement of feelings and motives. Based on this, he proposed the theory of utility called “marginal revolution,” in which psychological factors were introduced into economic analysis through utility functions when studying the determinants of demand. This is an essential start for understanding how psychological factors influence economic decisions and behaviour. After the Great Depression, Keynes J M [20] argued that expectation is a crucial determinant of output and employment. He differentiated short-term and long-term expectations and discussed how three critical psychological factors, the propensity to consume, liquidity preference, and expectations of future returns on capital, affect the total demand. This is the so-called theory of effective demand, in which expectation is crucial for individuals and firms. The work of Keynes J M [20] is one of the bases of modern economics. In short, Jevons W S [19] and Keynes J M [20] underscore the importance of human expectations to the economy as never before.

Although rational expectation (Muth [21]) has been widely used in modern economic research, with the development of behavioral economics, neuroeconomics, and neuroscience, the theoretical assumptions proposed by rational expectation, such as no systematic cognitive bias, consistent beliefs, and sufficient information, have been critiqued by some studies, such as Akerlof et al. [22] and Shiller [23]. Bounded rationality (Simon [24]) and imperfect rational expectations of information (Angeletos [25]) have gradually attracted academic attention. These studies have contributed significantly to understanding how psychological factors affect economic behaviour and the macroeconomy.

In recent years, research on confidence in the economy has mainly focused on economic growth (business cycle), fiscal and monetary policy, inflation, and prices.

For the nexus between confidence and economic growth (business cycle), existing studies have shown that consumer and entrepreneurial confidence are procyclical (Taylor and McNabb [7]). Consumer and entrepreneurial confidence improvements can stimulate domestic demand and promote economic growth (Liu et al. [26]). In other words, confidence positively correlates with economic growth (Guo and He [10]). As a result, many studies have incorporated confidence in economic growth forecasting and found that it improves the accuracy of predictions (Giannone et al. [27], Jiang and Li [28]). Nevertheless, some studies argue that although entrepreneurial confidence can influence macroeconomic fluctuations, consumer confidence cannot (Chen and Tang [13]). For the impact of confidence shocks on the macroeconomy, Zhuang et al. [9], Zhu and Deng [11], and Dees and Zimic [12] found that both expectation shocks and noise shocks have positive effects on main macroeconomic variables, and expectation shocks can explain a significant portion of economic growth fluctuations.

Fiscal and monetary policy are essential pillars of the macroeconomy in modern economics. How confidence affects the macroeconomy is a meaningful topic. Wang et al. [5] and Zhang and Sun [6] found that confidence plays a significant role in the transmission mechanism of monetary policy. Removing the bridging role of confidence could significantly reduce the effectiveness of monetary policy. Guo and He [10] and Li [29] found that the influence of confidence on the policy effect varies across different economic growth stages. Restoring confidence can amplify the policy effect and thus alter the policy effect multiplier. These results analyze the impact of confidence on a specific macroeconomic field, enriching the research on the relationship between confidence and the macroeconomy.

With the development of the internet, some studies attempt to utilize big data to study confidence. For example, Dong and Bollen [30], Liu and Li [31], and Yuan et al. [32] constructed internet consumer confidence indices with search engine data and explored their relationships with industrial value added, investment, consumption, and price. The results revealed that internet consumer confidence is a Granger cause of industrial value added and can explain approximately 20% of the variation in industrial value added. Changes in confidence also lead to investment, consumption, and price changes.

Recently, a growing body of studies on expectation has been using textual methods to extract the sentiment of media and then explore the effect of sentiment on the macroeconomy. For example, Larsen and Thorsrud [16] and Ardia et al. [33] showed that sentiment indicators computed by retrieving newspaper articles have predictive power for key economic variables, such as industrial production. Thorsrud’s [34] results suggest that the model with a daily business cycle index based on quarterly GDP growth and textual information is competitive with forecast combination systems and expert judgment. The results of a comparison study conducted by Ellingsen et al. [35] suggest that the news data contains information not captured by the hard economic indicators and that the news-based data are particularly informative for forecasting consumption developments.

Admittedly, the predictive power of sentiment indicators based on news articles prevails over survey data. However, it is worth noting that news articles are freely published and mainly convey the attitude of the media and authorities. In other words, the overall representativeness of the news is relatively poor. On the contrary, survey data is based on scientifically designed questionnaires and rigorous sampling survey theories. Thus, it is more representative of the population. Consequently, survey data is a better candidate for testing the nexus between confidence and the macroeconomy rather than forecasting the macroeconomy.

### 2.2. Decomposition of confidence

Furthermore, some researchers have further focused on the decomposition of confidence to explore the relationship between confidence and the macroeconomy. Harrison and Weder [36] and Barsky and Sims [37] proposed a decomposition of confidence into “fundamental confidence” and “animal spirits”. The former captures the part determined by macroeconomic fundamentals. Market participants estimate it based on macroeconomic information. The latter represents purely psychological or emotional factors that economic fundamentals cannot explain.

In fact, for confidence shocks, the literature has also adopted other terms, such as “sunspots” (Cass and Shell [38]), “self-fulfilling prophecies” (Azariadis [39]), and “waves of optimism and pessimism” (Chauvet and Guo [40]). Although these concepts have different names, they all refer to external uncertainties or shocks that are unrelated to economic fundamentals (Chen and Tang [13], Cass and Shell [38], Chauvet and Guo [40]). Among these concepts, “animal spirits” is the most widely used and well-known, so we adopt it in this paper.

The most popular research on “animal spirits” is conducted by Keynes [20]. He argued that investors make decisions not only based on mathematical expectations but also on irrational “animal spirits”. This assumption was adopted by Shleifer [41] when explaining why economic activities are aggregated. He concluded that people’s economic behaviour is influenced not only by economic factors but also by irrational factors such as emotions and beliefs. In recent years, some studies have shown that pessimistic consumers and entrepreneurs existed before several of the United States’ economic recessions, even when economic fundamentals were strong, suggesting that changes in consumer and investor perceptions are likely to be important factors influencing the business cycle (Dees and Zimic [12], Chauvet and Guo [40]). Moreover, the spread of pessimistic sentiment exacerbates crises and slows economic recovery (Assenza et al. [42]). Based on this, Nobel laureate Robert J. Shiller [23,43] systematically studied the impact of investor irrational sentiment on stock markets, bond markets, and real estate markets. Along with another Nobel laureate, George Akerlof [44], he attributed the concept of “animal spirits” as one of the root causes of the global financial crisis.

Similar studies have been done in China, but the conclusions are different. For example, Sui et al. [45] found that while “sunspot shocks” significantly impact endogenous variables, their role in transmitting fundamental shocks and fluctuating endogenous variables is weak, while Zhuang et al. [9] and Zhu et al. [11] showed that noise shocks have a positive effect on the macroeconomy.

### 2.3. Discussion about the existing research

In summary, existing studies expand the research on the relationship between macroeconomics and confidence. However, the nexus between macroeconomy and confidence is still controversial. Some studies suggest that the impact of confidence on the macroeconomy (Zhuang et al. [9], Dees and Zimic [12], Zhu and Deng [11]) is significant, and incorporating expectations retrieved from news articles or survey data into models can improve the accuracy of forecasting. Still, some studies provide opposite evidence, especially for China (Chen and Tang [13]).

There may be two reasons for this difference. One is the measurement error and sample size; the other is the limitation of the econometric model. Specifically, due to the late start of surveys on confidence, most early studies either adopted proxy indicators for confidence or used small samples. This leads to problems with sample size and measurement errors. Meanwhile, limited by econometric methods, most studies employed the same-frequency data models, which strictly require common-frequency data. The mixed-frequency variables are either replaced with proxy variables or aggregated through totalling or averaging. This transformation reduces the sample size but also results in the disorder of causal relationships between variables (Ghysels [17]). For instance, when using mixed-frequency and common-frequency data, Yuan and Wen [18] discovered a significant difference in the causal relationship between economic growth and consumer confidence.

Overall, the relationship between confidence and the macroeconomy and whether this relationship is robust in the context of mixed-frequency data needs more evidence, especially for the post-COVID era in China. Thus, this study adopts mixed-frequency data models and the newest sample to test the nexus between confidence and the macroeconomy. Mixed-frequency data methods can expand the sample size as much as possible. With the latest sample, the results can provide the newest and robust evidence of the relationship between confidence and the economy, especially in the context of mixed-frequency data and the post-COVID-19 era in China.

## 3. Variables and model

### 3.1. Variables and data

This study focuses on the relationship between confidence and the macroeconomy. Following Chen and Tang [13], Liu et al. [26], and Zhu and Deng [11], we measure confidence with the consumer confidence index (CCI). We depict the macroeconomy from five aspects: total output, development of the industrial entity, the financial market, price, and the labour market. Specifically, according to Harrison and Weder [36] and Chen and Tang [13], we use the GDP growth rate (GDP), the consumer price index (CPI), the purchasing managers’ index (PMI), the real estate climate index (RECI), the annual loan interest rate (R), the Shanghai stock composite index (Stock), and the urban unemployment rate (UE) to portray the macroeconomy. All monthly data ranges from January 2005 to June 2024. Accordingly, all quarterly data range from the first quarter of 2005 to the second quarter of 2024. The variables’ definitions, frequencies, and sources are summarized in Table 1.

**Table 1:** Variable definitions and data sources.

Variable	Definition	Frequency	Data Source	Transformation
CCI	Consumer Confidence Index	Monthly	CEIC	SA, difference
CPI	Consumer Price Index	Monthly	NBS	SA, detrending
PMI	Purchasing Manager Index	Monthly	NBS	SA, detrending
RECI	Real Estate Climate Index	Monthly	NBS	SA, difference
R	Annual Loan Interest Rate	Monthly	CEIC	SA
Stock	Shanghai Stock Composite Index	Monthly	CEIC	SA
GDP	GDP growth rate	Quarterly	NBS	detrending
UE	Urban Unemployment Rate	Quarterly	CEIC	detrending

Note: (1) NBS: National Data on the website of the National Bureau of Statistics of China (https://data.stats.gov.cn/english/easyquery.htm?cn=A01); (2) CEIC: China Premium Database of CEIC Data (https://insights.ceicdata.com.cn/insights/my). (3) SA: seasonal adjustment with X-13ARIMA-SEATS. All relevant data are collected in Supporting Information files S5 Data.

**Fig 2:**
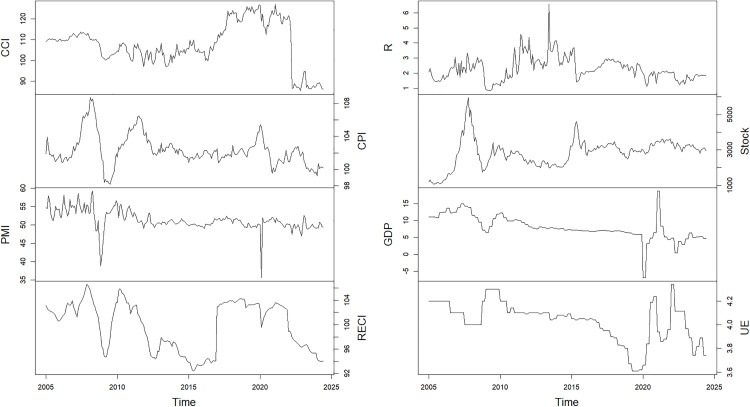
Time series of variables’ raw level. The X-axis indicates the time (quarter or month). The Y-axis indicates the raw levels of variables. See Table 1 for definitions of variables.

Fig 2 plots the raw level of these series. Intuitively, the monthly series shows a certain seasonality. Thus, we make seasonal adjustments to them with X-13ARIMA-SEATS. The quarterly GDP is the year-on-year growth rate, so there is no seasonality. The quarterly urban unemployment rate shows no apparent seasonality.

Lastly, non-stationary data may lead to misleading results in causality tests and RU-MIDAS. Thus, we conduct ADF tests before the formal analysis. The results are displayed in the S1 Table. It is shown that the levels of the consumer confidence index and the real estate market index are I(1). GDP, CPI, the purchasing managers’ index, and the urban unemployment rate have a deterministic trend. Thus, we make a difference (detrending) to the consumer confidence index and the real estate market index (GDP, CPI, the purchasing managers’ index, and urban unemployment rate), and adopt the data after difference and detrending in the following analysis.

### 3.2. Model specification

As mentioned above, performing temporal aggregation when modelling mixed-frequency data is common, especially in macroeconomic research. Specifically, macroeconomic data are often low-frequency due to statistical and economic costs. Annual and quarterly data are prevalent, while monthly, weekly, and daily data are scarce. Therefore, it is necessary to aggregate or average high-frequency data to low-frequency data in many scenarios. This approach not only leads to the loss of high-frequency information and decreases the sample size but also results in the distortion of causal relationships between variables (Ghysels [17]). To avoid this problem, mixed-frequency data models have been developed and applied rapidly in recent years. They have been widely used in economic and financial research, for example, Cui and Zhang [46] and Lu et al. [47]. The best advantage of these models is that they can incorporate the mixed-frequency data directly and consequently effectively maintain the actual relationships among the variables. These methods have attracted substantial attention because of their ability to utilize the information in unbalanced frequency datasets directly.

For our case, mixed-frequency data, such as the quarterly consumer confidence index and monthly CPI, will be used to test the relationship between confidence and the macroeconomy. Thus, mixed-frequency data models are better candidates. This paper uses mixed-frequency vector autoregression, mixed-frequency Granger causality tests, and reverse-constrained mixed-frequency data sampling to test this relationship. More details about these models can be found in Ghysels [48] and Foroni et al. [49].

#### 3.2.1. Mixed-frequency vector autoregressive models and mixed-frequency Granger causality test.

There are currently two forms of mixed-frequency vector autoregressive models (MF-VAR). The first form assumes that all variables in the mixed-frequency economic system share common high-frequency data-generating processes, while the low-frequency variables are latent and missing periodically. Thus, state space models can be used for modelling and analysis. For more details, see Mariano and Murasawa [50] and Lu et al. [47]. However, the parameter estimated by this approach cannot be directly used for the Granger causality test (Ghysels et al. [17], Yuan and Wen [18]). Therefore, Ghysels et al. [17] proposed another form. This approach is more appropriate for our case.

Let $x_{H}$ and $x_{L}$ denote high- and low-frequency stationary variables, respectively. The ratio of sampling frequencies is m. In this paper, $x_{H}$ and $x_{L}$ are monthly and quarterly variables in Table 1. Accordingly, m=3. Let $\tau_{L}\in \{0,...,T\}$ denote the time mark of low-frequency data. $x_{H}(\tau_{L},j)$, j∈{1,...,m} represents the *j-*th high-frequency observation of $x_{L}$. For example, if j=3, $x_{H}(\tau_{L},3)$ marks the third-month value of the high-frequency consumer confidence index in a specific quarter $\tau_{L}$. After standardizing, we can stack the data into an (m+1)×T mixed-frequency matrix $X(\tau_{L})=(x_{H}{\left(\tau_{L},\text{1}\right)}^{\prime },\;\cdots ,\;x_{H}\left(\tau_{L},m\right),x_{L}(\tau_{L})\prime )$. Assuming it follows a VAR(*p*) process, an MF-VAR(*p*) model can be established as


$$\begin{array}{c} \left[\begin{array}{c} x_{H}\left(\tau_{L},\text{1}\right)\\ \vdots \\ x_{H}\left(\tau_{L},m\right)\\ x_{L}\left(\tau_{L}\right) \end{array}]=\sum_{i=\text{1}}^{p}[\begin{array}{cccc} a_{\text{11},i} & \cdots & a_{\text{1}m,i} & c_{(i-\text{1})m+\text{1}}\\ \vdots & \ddots & \vdots & \vdots \\ a_{m\text{1},i} & \cdots & a_{mm,i} & c_{im}\\ b_{im} & \cdots & b_{(i-\text{1})m+\text{1}} & a_{i} \end{array}]\times [\begin{array}{c} x_{H}\left(\tau_{L}-i,\text{1}\right)\\ \vdots \\ x_{H}\left(\tau_{L}-i,m\right)\\ x_{L}\left(\tau_{L}-i\right) \end{array}]+[\begin{array}{c} \varepsilon_{H}\left(\tau_{L},\text{1}\right)\\ \vdots \\ \varepsilon_{H}\left(\tau_{L},m\right)\\ \varepsilon_{L}\left(\tau_{L}\right) \end{array}\right] \end{array}$$ (1)

As a result, conducting a Granger causality test for confidence and the macroeconomy is equivalent to testing the parameters in Equation (1). For example, the null hypothesis that consumer confidence (GDP) is not a Granger cause of GDP (consumer confidence) cannot be rejected if and only if $b_{im}=...=b_{(i-\text{1})m+\text{1}}=\text{0}$ ($c_{(i-\text{1})m+\text{1}}=...=c_{im}=\text{0}$).

The reduced form of Equation (1) is $X(\tau_{L})=\sum_{i=\text{1}}^{p}A_{i}X\left(\tau_{L}-i\right)+\varepsilon \left(\tau_{L}\right)$. To test the multiperiod relationships between the high-frequency and low-frequency variables, such as consumer confidence and GDP, the model can be expanded to the MF-VAR(p,h) model, where h refers to the number of periods:


$$\begin{array}{c} X\left(\tau_{L}+h\right)=\sum_{i=\text{1}}^{p}A_{i}^{\left(h\right)}X\left(\tau_{L}-i\right)+\varepsilon^{\left(h\right)}\left(\tau_{L}\right) \end{array}$$ (2)

where $A_{i}^{\left(\text{1}\right)}=A_{i}$, $A_{i}^{\left(j\right)}=A_{i+j-\text{1}}+\sum_{l=\text{1}}^{j-\text{1}}A_{j-l}A_{i}^{\left(l\right)}(j\geq \text{2})$, and $\varepsilon^{\left(h\right)}(\tau )=\sum_{i=\text{0}}^{h-1}\phi_{i}\varepsilon (\tau -k)$. Testing the causal relationships between mixed-frequency variables over *h* periods is equivalent to testing whether the following constraints hold:


$$H_{\text{0}}(h):Rvec[B(h)]=r$$

where R is a selection matrix with full row rank and $B(h)=[A_{\text{1}}^{\left(h\right)},...,A_{p}^{\left(h\right)}{]}^{\prime }$ is the coefficient of the MF-VAR(*p, h*) model. r is the constraint vector. The test statistic can be constructed by


$$\begin{array}{c} W=(T-h+\text{1}){\left(Rvec\left[\hat{B}(h)\right]-r\right)}^{\prime }\times (R{\hat{\Sigma }}_{B_{p}(h)}R^{\prime {)}^{-\text{1}}}\times \left(Rvec\left[\hat{B}(h)\right]-r\right) \end{array}$$ (3)

where $\hat{B}(h)$ is the estimated ordinary least squares coefficients. ${\hat{\Sigma }}_{B_{p}(h)}$ is the consistent estimator of variance.

Under the null hypothesis, the *p-*value of the statistic can be calculated through Bootstrap by


$$\begin{array}{c} p_{N}(W)=\frac{\text{1}}{N+\text{1}}[\text{1}+\sum_{h=\text{1}}^{N}I(W_{h}\geq W)] \end{array}$$ (4)

where N is the sample size. I(·) is the indicator function. At a given significance level α, if $p_{N}(W)\leq \alpha$, the null hypothesis is rejected, suggesting the existence of Granger causality among the variables.

In our case, after difference and detrending, we can stack series in a mixed-frequency matrix (omitting the time mark):


$$X\left(\tau_{L}\right)=(CCI\_1,CP\text{I}\_\text{1},PM\text{I}\_\text{1},REC\text{I}\_\text{1},\text{R}\_\text{1},Stock\_\text{1},CCI\_2,CPI\_2,PMI\_2,RECI\_2,\text{R}\_\text{2},Stock\_2,$$

$$\begin{array}{c} CCI\_3,CP\text{I}\_\text{3},PM\text{I}\_\text{3},REC\text{I}\_\text{3},\text{R}\_\text{3},Stock\_3,GDP,\;UE{)}^{\prime } \end{array}$$

where CCI_1, CCI_2, and CCI_3 indicate the first, second and third monthly consumer confidence index in a quarter $\tau_{L}$, respectively. The same for other variables. Based on this matrix, the MF-VAR model displayed in Equation (2) can be established. Then, the Wald statistic and corresponding *p-*value defined by Equations (3) and (4) can be calculated with Bootstrap to test the Granger causality among mixed-frequency variables. According to this statistic, we can test a series of null hypotheses, such as that consumer confidence is not the Granger cause of GDP, and GDP is not the Granger cause of consumer confidence.

#### 3.2.2. The reverse unconstrained mixed data sampling model.

To utilize mixed-frequency data more effectively, Ghysels et al. [51] proposed the MIxed DAta Sampling (MIDAS) model based on distributed lag models. A linear combination of high-frequency variables explains a low-frequency variable in this model. This model requires a form of nonlinear least squares estimation, which increases the computational costs substantially in specifications with more than one high-frequency explanatory variable. Thus, it works well only in a univariate context where the frequency of the dependent variable is low and that of the independent variable is high, for example, quarterly to monthly. To avoid this limitation, Foroni et al. [49] introduced the reverse unconstrained mixed data sampling model (RU-MIDAS). In our case, the dependent variable is the high-frequency consumer confidence index, whereas the independent variables contain low-frequency macroeconomic variables such as GDP and urban unemployment rate. Therefore, RU-MIDA is more appropriate.

According to Foroni et al. [49], a RU-MIADS model can be represented as


$${\tilde{c}}_{\text{0}}(L)x_{\tau }=\gamma_{\text{0}}(L)\omega (L)c(L)x_{\tau }{=\gamma }_{\text{0}}(L)d(L)\omega (L)y_{\tau }^{*}+\gamma_{\text{0}}(L)\omega (L)e_{x_{\tau }}=g_{\text{0}}(Z)y_{t}+{\tilde{\gamma }}_{\text{0}}(L)e_{x_{\tau }}$$ (5)

where $x_{\tau }$ and $y_{t}$ are high- and low-frequency variables, respectively. $y_{\tau }^{*}$ is the potential high-frequency counterpart of $y_{t}$. L is the high-frequency lag operator, i.e., $L^{j}x_{\tau }=x_{\tau -j/m}$. m is the ratio of sampling frequencies of $y_{t}$ and $x_{\tau }$. $c(L)=I-c_{\text{1}}L-\cdots -c_{p}L^{p}$. $d(L)=d_{\text{1}}L+d_{\text{2}}L+\cdots +d_{p}L^{p}$. $\omega (L)=\omega_{\text{0}}+\omega_{\text{1}}L+...+\omega_{m-\text{1}}L^{m-\text{1}}$ is a high-frequency lag operator polynomial that characterizes the aggregation mechanism between low-frequency $y_{t}$ and $y_{\tau }^{*}$. That is, $y_{t}=\omega (L)y_{\tau }^{*}$. $\gamma_{\text{0}}(L)$ is a high-frequency lag operator polynomial satisfying the condition that $\gamma_{\text{0}}(L)d(L)$ contains only $L^{m}$. $e_{x_{\tau }}$ is white noise.

In practice, though, the parameters of Equation (5) are unknown, meaning that ${\tilde{\gamma }}_{\text{0}}(L)$ cannot be determined precisely. Hence, empirically, we will use approximate RU-MIDA models based on linear lag polynomials, such as


$${\tilde{a}}_{\text{i}}(L)x_{\tau }={\tilde{b}}_{\text{i}}\left(L^{k+i}\right)y_{\tau }+\xi_{\tau }$$

$$\tau =0+\frac{i}{m},\;1+\frac{i}{m},\;2+\frac{i}{k},\;\cdots ,$$ (6)

i=1, 2, ⋯, m

where the orders of ${\tilde{a}}_{\text{i}}(L)$ and ${\tilde{b}}_{\text{i}}\left(L^{k+i}\right)$, $a_{i}$ and $b_{i}$, respectively, are large enough to make $\xi_{it}$ white noise.

The estimation of the RU-MIDAS is straightforward since it is a linear model. Hence, the parameters of the model in Equation (6) can be estimated by ordinary least squares for each value of i, and the lag orders $a_{i}$ and $b_{i}$ can be selected using standard information criteria such as the Akaike information criterion and Bayesian information criterion. Moreover, the RU-MIDAS model in Equation (6) can be extended easily to allow additional high-frequency or low-frequency explanatory variables.

In our case, to decompose the confidence index, referring to Harrison and Weder [36] and Chen and Tang [13], we set the model as


$$\begin{array}{c} C{CI}_{t+\frac{i}{m}}=c_{t+\frac{i}{m}}+\sum_{j=\text{1}}^{l_{\text{0}}}\alpha_{i,j}^{\text{0}}{CCI}_{t+\frac{i}{m}-\frac{j}{m}}+\sum_{j=\text{0}}^{l_{\text{1}}}\alpha_{i,j}^{\text{1}}{CPI}_{t+\frac{i}{m}-\frac{j}{m}}+\sum_{j=\text{0}}^{l_{\text{2}}}\alpha_{i,j}^{2}{PMI}_{t+\frac{i}{m}-\frac{j}{m}} \end{array}$$

$$\begin{array}{c} +\sum_{j=\text{0}}^{l_{\text{3}}}\alpha_{i,j}^{\text{3}}{RECI}_{t+\frac{i}{m}-\frac{j}{m}}+\sum_{j=\text{0}}^{l_{\text{4}}}\alpha_{i,j}^{\text{4}}R_{t+\frac{i}{m}-\frac{j}{m}}+\sum_{j=\text{0}}^{l_{\text{5}}}\alpha_{i,j}^{\text{5}}{Stock}_{t+\frac{i}{m}-\frac{j}{m}} \end{array}$$

$$\begin{array}{c} +\sum_{k=\text{0}}^{p_{\text{1}}}\beta_{i,k}^{\text{1}}{GDP}_{t-k}+\sum_{k=\text{0}}^{p_{\text{2}}}\beta_{i,k}^{\text{2}}{UE}_{t-k}+\varepsilon_{t+\frac{i}{m}} \end{array}$$ (7)

where m=3 is the ratio of sampling frequencies. i=1,2,…,m is the *i-*th month within a certain quarter. $\alpha_{i,j}^{q}(q=0,1,2,\cdots ,5$) and $\beta_{i,k}^{s}(s=1,2)$ are the regression coefficients of variables and their lagged terms, respectively. $l_{q}(q=0,1,2,\cdots ,5))$ and $p_{s}(s=1,2)$ represent the optimal lag orders for low-frequency and high-frequency variables, respectively. $c_{t+i/m}$ and $\varepsilon_{t+i/m}$ are constant and random disturbances, respectively.

## 4. Empirical results

### 4.1. Descriptive statistics and correlation analysis

Table 2 presents the descriptive statistics of the variables. Combining Fig 2, we can find that the variables exhibit similar trends. This is preliminary evidence about the interaction between confidence and the macroeconomy. Specifically, due to the impacts of the global financial crisis and the COVID-19 pandemic, these variables significantly decreased in 2008 and 2020, though the consumer confidence index dropped sharply in April 2022.

**Table 2:** Descriptive statistics.

Variable	N	Mean	SD	Min	Max
CCI_1	77	−0.2659	4.6081	−31.9425	8.4228
CCI_2	77	−0.2989	4.4689	−29.1018	8.1053
CCI_3	77	−0.3026	4.0206	−23.2006	7.8243
CPI_1	77	0.0187	1.8261	−4.6541	5.5114
CPI_2	77	−0.0062	1.8299	−4.5609	5.6246
CPI_3	77	0.0091	1.7433	−4.6171	5.2403
PMI_1	77	−0.0114	2.1483	−8.2748	6.1987
PMI_2	77	−0.0191	2.6565	−14.0644	3.4881
PMI_3	77	−0.0480	2.0577	−12.0174	4.9848
RECI_1	77	−0.1211	1.6643	−3.8409	8.9581
RECI_2	77	−0.1184	1.7453	−4.0384	8.7835
RECI_3	77	−0.1135	1.7462	−4.5957	9.0909
R_1	77	2.2875	0.7465	0.9590	4.3561
R_2	77	2.2432	0.6665	0.8902	4.0123
R_3	77	2.2984	0.8662	0.8774	6.6118
Stock_1	77	2845.9305	800.6184	1105.3657	5979.5659
Stock_2	77	2831.6432	765.4755	1003.9506	5326.1113
Stock_3	77	2871.4652	765.6866	1133.9948	5630.2824
GDP	77	0.0152	2.5466	−12.5107	13.5332
UE	77	−0.0001	0.1374	−0.3393	0.4373

Note: This table reports the descriptive statistics of transformed variables. N, Mean, SD, Min, and Max refer to the number, standard deviation, minimum, and maximum of observations, respectively. CCI_1, CCI_2, and CCI_3indicate the first, second and third monthly consumer confidence index in a quarter $\tau_{L}$, respectively—the same for other variables. See Table 1 for definitions of variables.

Before the formal analysis, we calculated pairwise Pearson and Spearman coefficients. The results (S2 Table) show that the purchasing managers’ index, the real estate climate index, interest rate, Shanghai stock composite index, and GDP (CPI, urban unemployment rate) are positively (negatively) correlated with the consumer confidence index. This is further evidence of the close relationship between confidence and the macroeconomy.

### 4.2. Granger causality tests

According to the AIC, the optimal lag order for the model displayed in Equation (2) is 2. The Wald statistic and corresponding p-values defined by Equations (3) and (4) are calculated with the Bootstrap method. The replication is set to 1000. The results are presented in Table 3.

**Table 3:** Results of the mixed-frequency Granger causality test.

Null hypothesis(H0)	*p-*value	Reject H0	Null hypothesis(H0)	*p-*value	Reject H0
CCI↛CPI	0.0150	Yes	CPI↛CCI	0.9171	No
CCI↛PMI	0.1858	No	PMI↛CCI	0.3017	No
CCI↛RECI	0.4595	No	RCEI↛CCI	0.1019	Yes
CCI↛R	0.8711	No	R↛CCI	0.7362	No
CCI↛Stock	0.1459	No	Stock↛CCI	0.7163	No
CCI↛GDP	0.7143	No	GDP↛CCI	0.0380	Yes
CCI↛UE	0.4076	No	UE↛CCI	0.0509	Yes

Note: CCI↛CPI represents that CCI is not a Granger cause of CPI. The same applies to others. See Table 1 for definitions of variables.

It is found that, at the 10% significance level, the real estate climate index, GDP, and urban unemployment rate are Granger causes of consumer confidence, suggesting that changes in the real estate market, GDP, and urban unemployment rate will lead to changes in consumer confidence. Meanwhile, consumer confidence is a Granger cause of the CPI, indicating an intuitive conclusion that consumer confidence significantly influences consumer goods prices. Lastly, we cannot reject the null hypothesis that consumer confidence is not a Granger cause of GDP and urban unemployment rate in our sample. The reason may be a nonlinear Granger causality relationship between confidence and GDP and urban unemployment rate (Yuan et al. [32]).

### 4.3. The decomposition of confidence

Based on the AIC, the optimal lag orders in Equation (6) are determined. The estimation results of RU-MIDAS are presented in Table 4. As stated in Table 1, the consumer confidence index and real estate climate index are first-order differenced. The results show that the lagged changes in consumer confidence can explain itself significantly. Positive changes in the previous consumer confidence will lead to negative changes in the current period. In other words, the changes in consumer confidence proceed negatively despite the raw levels growing on the whole, despite the level shift effect of COVID-19 (see Fig 1). Second, CPI has a significant negative impact on changes in consumer confidence, indicating that as prices grow, the changes in consumer confidence will decline. This means that inflation is undesired among consumers. Third, changes in the real estate market positively impact changes in consumer confidence in the current and lagged periods, suggesting that the real estate market’s prosperity will significantly boost consumer confidence. This result is consistent with the basic facts – China’s macroeconomy’s rapid development has been driven considerably by the real estate market in recent years. Lastly, the purchasing managers’ index, interest rate, and Shanghai stock composite index are hardly essential factors affecting confidence. The reason is that these variables have progressed so steadily in recent years that consumers think they will not fluctuate significantly and become insensitive to these variables.

**Table 4:** Estimation results of RU-MIDAS.

	CCI_*i* (i=1)	CCI_*i* (i=2)	CCI_*i* (i=3)
L(CCI_*i*, 1)	−0.2783**	−0.1929*	−0.0562
	(0.1174)	(0.1106)	(0.1110)
L(CCI_*i*, 2)	−0.2934**	−0.1588	−0.2449**
	(0.1161)	(0.1057)	(0.1098)
L(CCI_*i*, 3)	−0.2173**	−0.1982*	−0.4712***
	(0.1056)	(0.1055)	(0.1145)
CPI_*i*	−0.6704	−1.2083**	−0.5941*
	(0.5923)	(0.4688)	(0.3173)
L(CPI_*i*, 1)	−1.1737	0.8178	
	(0.7773)	(0.5130)	
L(CPI_*i*, 2)	1.3275**		
	(0.5484)		
PMI_*i*	0.0870	0.1720	0.3143
	(0.2477)	(0.2292)	(0.2245)
L(PMI_*i*, 1)			−0.3506
			(0.2337)
RECI_*i*	0.3104	0.3056	0.8068***
	(0.2958)	(0.2961)	(0.2596)
L(RECI_*i,* 1)	1.3890***	0.9120***	0.4199
	(0.3082)	(0.2914)	(0.2562)
L(RECI_*i*, 2)	−0.1375		0.1955
	(0.3105)		(0.2519)
L(RECI_*i*, 3)	0.8089***		0.4572*
	(0.3024)		(0.2456)
R_*i*	0.8870	0.2846	0.0289
	(0.9232)	(1.1617)	(0.5569)
L(R_*i*, 1)	1.1732	1.6061	1.5594***
	(0.8548)	(1.1353)	(0.5289)
Stock_*i*	0.0015	0.0020	−0.0005
	(0.0010)	(0.0013)	(0.0006)
L(Stock_*i*, 1)	−0.0015	−0.0024*	
	(0.0011)	(0.0013)	
GDP	0.2758	0.4681*	0.3325*
	(0.2130)	(0.2373)	(0.1868)
L(GDP, 1)			0.4803**
			(0.1955)
L(GDP, 2)			−0.3973**
			(0.1699)
UE	8.1152	5.0223	7.3168
	(5.4070)	(5.5568)	(4.9400)
L(UE, 1)	−31.7133***	−25.5285***	−28.8266***
	(7.0206)	(7.1357)	(6.4573)
L(UE, 2)	12.3094**	11.3568**	9.0849*
	(5.6464)	(5.5759)	(4.9675)
Observations	74	74	74
Adjusted R^2^	0.4389	0.3758	0.4745
F Statistic	4.0465*** (df = 19; 55)	3.7843*** (df = 16; 58)	4.5162*** (df = 19; 55)
White heteroskedasticity test	74 [0.4453]	74 [0.4453]	74 [0.4453]
Ramsey RESET test	19.7200 [0.0001]	15.34 [0.0001]	11.97 [0.0001]
Mean variance inflation factor	3.4800	3.10	2.33

Note: (1) This table reports the estimation results of RU-MIDAS for i=1, 2, 3 in Equation (6). L(CCI_*i*, 1) and L(GDP, 1) represent the lagged CCI_*i* and UE, respectively. The same applies to the other variables. See Table 1 for definitions of variables. (2) *t-*statistic in parentheses and *p-*value in brackets. * p < 0.1, ** p < 0.05, ***p < 0.01.

For low-frequency variables, GDP positively affects changes in consumer confidence, suggesting the evident and close nexus between consumer confidence and the macroeconomy, which is supported by most existing research. Lagged urban unemployment rate significantly negatively affects the change in consumer confidence. This is because employment and, thus, income correlate with confidence directly. These conclusions are mainly aligned with the mixed-frequency Granger causality test and most existing literature, such as Soric et al. [14].

### 4.4. The effect of animal spirits on the macroeconomy

**Fig 3:**
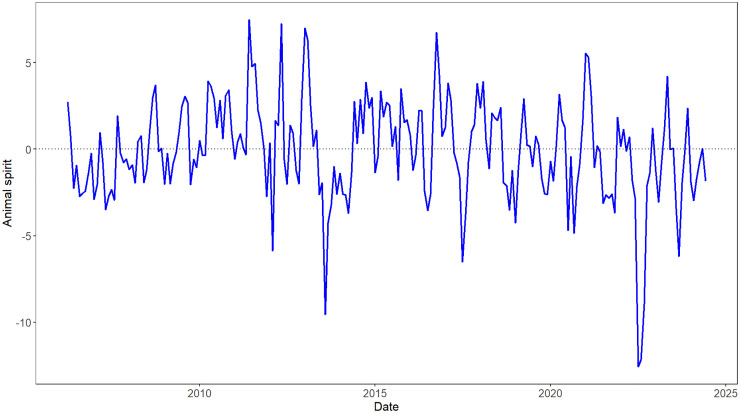
Animal spirits series. The X-axis indicates the date (month), and the Y-axis indicates the level of animal spirits.

The residual component that cannot be explained by macroeconomic fundamentals in the decomposition is the “animal spirits” (Fig 3). It fluctuates around zero and reaches a minimum in July 2022 due to the sluggish economic recovery following the COVID-19 pandemic. Additionally, it suffers from two fluctuation periods: one from early 2010 to the end of 2014, attributed to the spread of the financial crisis, and another from early 2017 to the end of 2022, influenced by Sino-US trade disputes and global COVID-19.

**Fig 4:**
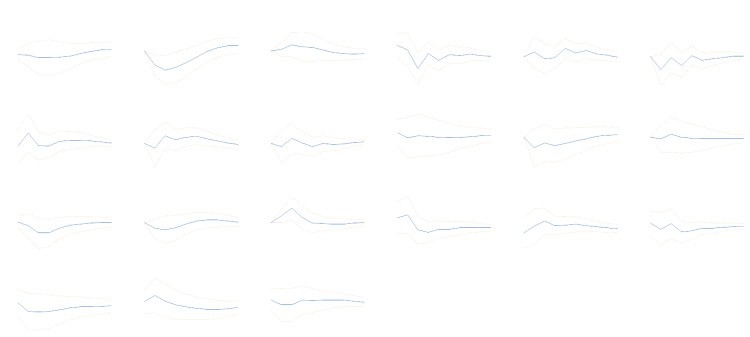
Impulse response functions of the macroeconomic variables to “animal spirits”. See Table 1 for definitions of variables. The horizontal axis tells the number of periods. The vertical axis indicates the values of the orthogonalized impulse response functions. The dotted lines indicate 95% confidence intervals.

We performed impulse response analysis to explore the relationship between “animal spirits” and the macroeconomy. Fig 4 shows the graph of the orthogonalized impulse response functions. It is found that most macroeconomic variables respond to the innovation from animal spirits in step 2 and beyond, indicating that the macroeconomy reacts to shocks from animal spirits slowly. And more importantly, most error bands include zero, meaning that most impulse responses are statistically insignificant. This means that shocks from animal spirits did not lead to noticeable fluctuations in China’s macroeconomy. This is because we have extracted the fundamental confidence from consumer confidence. The remaining is a purely irrational component. And whether under the rational expectation (Muth [21]), the bounded rationality (Simon [24]), or the imperfect rational expectations of information (Angeletos [25]), purely irrational expectations will not be the dominant factor in economic development solely.

### 4.5. Robustness check

#### 4.5.1. Replace consumer confidence index with economist confidence index.

In this section, we replace the dependent variable – consumer confidence index with the economist confidence index to test the reliability of the results. The Economist confidence index is a monthly confidence index published by YICAI (https://www.yicai.com/news/101575561.html), a special media focusing on the economy in China. This index reflects the confidence of more than 200 chief economists in China. Following the procedure above, the ADF tests (S1 Table) show that the level of the economist confidence index is stationary. The results of RU-MIDAS (Equation (6) with the economist confidence index on the left-hand side) are collected in the S3 Table.

It is found that economists’ confidence is also significantly autocorrelated. The lagged terms of the economist confidence index have strong explanatory power on their own. Second, positive changes in CPI, the purchasing managers’ index and the real estate climate index will also cause positive changes in economists’ confidence. The effects of interest rate and the Shanghai stock market on economists’ confidence are still insignificant. The impact of GDP and urban unemployment rate is positive and negative, respectively. These results align with the decomposition of the consumer confidence index.

#### 4.5.2. The influence of the global financial crisis and COVID-19.

The sample period in this study is from January 2005 to Jun 2024. This means that this study covers the global financial crisis and the COVID-19 pandemic period, which may affect the results. To check the robustness of the conclusions, we performed the analysis by comparing normal and non-tranquil periods. Specifically, based on the fact that, on September 14th, 2008, Lehman Brothers filed for bankruptcy and Merrill Lynch announced its acquisition by Bank of America which is widely recognized as the sign of the outbreak of the global financial crisis, we divided the first non-tranquil periods which spans from September, 2008 to July, 2010 in which the consumer confidence index returned to what it was before the financial crisis. Similarly, based on another fact that, on January 23, 2020, the Wuhan Government announced that all traffic in this city was suspended, which is widely recognized as the start of the COVID-19 pandemic period in China, we divided the second non-tranquil periods which spans from January, 2020 to June, 2024 in which the consumer confidence index did not return to what it was before the COVID-19 pandemic but the sample is end.

According to the division of non-tranquil periods, we split the sample into three subsamples: including the global financial crisis but excluding COVID-19, including COVID-19 but excluding the global financial crisis, and excluding both the global financial crisis and COVID-19 to test the influence of the global financial crisis and COVID-19 on the relationship between confidence and the macroeconomy.

The results of RU-MIDAS using the three subsamples above are displayed in S4 Table. It is found that most results are in accordance with Section 4.3. For example, consumer confidence is autocorrelated significantly in all subsamples; CPI has a negative impact on changes in consumer confidence in most cases; Changes in the real estate market have a positive impact on changes in consumer confidence; the purchasing managers’ index, interest rate, and Shanghai stock market have a significant influence on consumer confidence only in a few sub-models. This suggests that the research conclusion above is robust.

## 5. Conclusion and discussion

This paper first analyzes the theoretical relationship between confidence and the macroeconomy. Based on this, it adopts mixed-frequency Granger causality tests to examine the Granger causality between consumer confidence and the macroeconomy. Then, the reverse unconstrained mixed-frequency data sampling model is applied to decompose confidence into “fundamental confidence”, which can be explained by macroeconomic fundamentals, and “animal spirits”, which cannot be explained by macroeconomic fundamentals. Finally, a battery of robustness tests is performed to demonstrate that the main findings remain consistent.

The results show that changes in the real estate market, GDP, and urban unemployment rate are Granger causes of consumer confidence, and in reverse, consumer confidence is a Granger cause of the CPI. Meanwhile, GDP and the real estate market (CPI and urban unemployment rate) have a significant positive (negative) impact on consumer confidence, while the conditions of industrial production, interest rate, and stock market do not. This implies that the consumer confidence is an important factor in the China’s macroeconomy and the conditions of prices, real estate market and labor market will influence the consumer confidence significantly but the consumers are insensitive to industrial production, interest rate and stock market because these variables progressed so steadily in recent years in China that consumers think there will not be surprising fluctuations in these variables. Second, the “animal spirits” extracted from consumer confidence cannot lead to noticeable fluctuations in China’s macroeconomy. This suggests that the “animal spirits” will not dominate economic growth, even though they affect the macroeconomy slightly and inevitably. The results are robust after replacing the dependent variable and thinking about the influence of the global financial crisis and the COVID-19 pandemic.

These conclusions align with most existing research and enrich the studies on confidence and the economy. However, mixed-frequency data models with the latest sample improved the test’s performance and thus helped explore the substantive relationship between confidence and the economy as much as possible. A battery of robustness tests also provides more evidence about the nexus between confidence and the economy in the post-COVID-19 era in China.

Admittedly, limited by the estimation feasibility and computational complexity, we just adopted the monthly and quarterly data in the models because overlarge sampling frequency differences will cause the sparsity of observation and thus the difficulty of estimation for the models. Therefore, one possible extension in the future is to utilize the variables sampled at more frequencies directly in the mixed-frequency data model.

## Supporting information

S1 Table Results of the ADF test. (DOCX)

S2 Table Correlation matrix. (DOCX)

S3 Table Estimation results of the economist confidence index decomposition. (DOCX)

S4 Table Results of RU-MIDAS with subsamples. (DOCX)

S5 Data The data used in this paper. (XLSX)
