# Supplementary material for: Confidence, animal spirits, and the macroeconomy in China: Based on mixed-frequency data models
Source: PLoS One. 2025 Sep 19;20(9):e0332909. doi: 10.1371/journal.pone.0332909 (PMC12448974; doi:10.1371/journal.pone.0332909)
Supplement: S1 Table — (DOCX) [file pone.0332909.s001.docx]

## S1 Table

Results of the ADF test

| Variables | Category | Form | Statistics | CV5 | *p*-value | Trend | Trend_p | Constant | Constant_p |
| --- | --- | --- | --- | --- | --- | --- | --- | --- | --- |
| CCI_1 | Raw | (C, T, 0) | -1.5643 | -3.4692 | 0.798 | -0.0185 | 0.4328 | 9.1998 | 0.1111 |
|  |  | (C, 0, 0) | -1.5529 | -2.8996 | 0.5016 |  |  | 8.3908 | 0.1381 |
|  |  | (0, 0, 0) | -0.6479 | -1.9451 | 0.4334 |  |  |  |  |
|  | Difference | (C, T, 0) | -9.2878 | -3.47 | 0 | -0.0182 | 0.4593 | 0.4049 | 0.7148 |
|  |  | (C, 0, 0) | -9.2873 | -2.9001 | 0 |  |  | -0.313 | 0.56 |
|  |  | (0, 0, 0) | -9.3098 | -1.9451 | 0 |  |  |  |  |
| CCI_2 | Raw | (C, T, 0) | -1.4355 | -3.4692 | 0.8426 | -0.0191 | 0.404 | 8.3738 | 0.1414 |
|  |  | (C, 0, 0) | -1.4212 | -2.8996 | 0.5677 |  |  | 7.5314 | 0.1775 |
|  |  | (0, 0, 0) | -0.7143 | -1.9451 | 0.4041 |  |  |  |  |
|  | Difference | (C, T, 0) | -8.788 | -3.47 | 0 | -0.0194 | 0.4191 | 0.4519 | 0.6751 |
|  |  | (C, 0, 0) | -8.7708 | -2.9001 | 0 |  |  | -0.3105 | 0.5527 |
|  |  | (0, 0, 0) | -8.7883 | -1.9451 | 0 |  |  |  |  |
| CCI_3 | Raw | (C, T, 1) | -1.5909 | -3.47 | 0.7876 | -0.0157 | 0.455 | 8.5262 | 0.1057 |
|  |  | (C, 0, 1) | -1.6073 | -2.9001 | 0.474 |  |  | 7.9654 | 0.1252 |
|  |  | (0, 0, 1) | -0.6922 | -1.9451 | 0.4138 |  |  |  |  |
|  | Difference | (C, T, 0) | -7.2817 | -3.47 | 0 | -0.0162 | 0.4454 | 0.384 | 0.6882 |
|  |  | (C, 0, 0) | -7.2614 | -2.9001 | 0 |  |  | -0.255 | 0.5826 |
|  |  | (0, 0, 0) | -7.2743 | -1.9451 | 0 |  |  |  |  |
| CPI_1 | Raw | (C, T, 9) | -4.0881 | -3.4773 | 0.0103 | -0.0245 | 0.0006 | 67.4502 | 0.0001 |
|  |  | (C, 0, 9) | -1.8717 | -2.9048 | 0.3435 |  |  | 22.4294 | 0.0668 |
|  |  | (0, 0, 8) | -0.3934 | -1.9456 | 0.5387 |  |  |  |  |
|  | Detrending | (C, T, 9) | -4.0881 | -3.4773 | 0.0103 | -0.0071 | 0.1274 | 0.4331 | 0.0569 |
|  |  | (C, 0, 9) | -3.845 | -2.9048 | 0.004 |  |  | 0.118 | 0.2026 |
|  |  | (0, 0, 9) | -3.604 | -1.9457 | 0.0005 |  |  |  |  |
| CPI_2 | Raw | (C, T, 8) | -3.6394 | -3.4763 | 0.0337 | -0.0248 | 0.0019 | 66.7624 | 0.0006 |
|  |  | (C, 0, 8) | -1.6688 | -2.9042 | 0.4424 |  |  | 21.2927 | 0.1012 |
|  |  | (0, 0, 8) | -0.4517 | -1.9456 | 0.5157 |  |  |  |  |
|  | Detrending | (C, T, 8) | -3.6394 | -3.4763 | 0.0337 | -0.0064 | 0.2065 | 0.3739 | 0.1299 |
|  |  | (C, 0, 8) | -3.4775 | -2.9042 | 0.0115 |  |  | 0.0913 | 0.3746 |
|  |  | (0, 0, 8) | -3.3665 | -1.9456 | 0.001 |  |  |  |  |
| CPI_3 | Raw | (C, T, 9) | -3.8197 | -3.4773 | 0.0213 | -0.0213 | 0.0016 | 60.2624 | 0.0003 |
|  |  | (C, 0, 9) | -1.8096 | -2.9048 | 0.3729 |  |  | 18.7751 | 0.0763 |
|  |  | (0, 0, 8) | -0.4513 | -1.9456 | 0.5159 |  |  |  |  |
|  | Detrending | (C, T, 9) | -3.8197 | -3.4773 | 0.0213 | -0.0048 | 0.2423 | 0.2968 | 0.1381 |
|  |  | (C, 0, 9) | -3.6542 | -2.9048 | 0.007 |  |  | 0.0838 | 0.3023 |
|  |  | (0, 0, 9) | -3.5013 | -1.9457 | 0.0007 |  |  |  |  |
| PMI_1 | Raw | (C, T, 4) | -4.9142 | -3.4726 | 0.0008 | -0.052 | 0.0005 | 44.5586 | 0 |
|  |  | (C, 0, 11) | -2.029 | -2.9062 | 0.274 |  |  | 16.2543 | 0.0521 |
|  |  | (0, 0, 11) | -1.6154 | -1.9458 | 0.0996 |  |  |  |  |
|  | Detrending | (C, T, 4) | -4.9142 | -3.4726 | 0.0008 | 0.0003 | 0.9752 | -0.0124 | 0.9773 |
|  |  | (C, 0, 4) | -4.952 | -2.9018 | 0.0001 |  |  | -0.0004 | 0.9985 |
|  |  | (0, 0, 4) | -4.9922 | -1.9453 | 0 |  |  |  |  |
| PMI_2 | Raw | (C, T, 0) | -6.823 | -3.4692 | 0 | -0.0402 | 0.0082 | 40.7692 | 0 |
|  |  | (C, 0, 0) | -6.0096 | -2.8996 | 0 |  |  | 32.6299 | 0 |
|  |  | (0, 0, 4) | -0.4257 | -1.9453 | 0.5262 |  |  |  |  |
|  | Detrending | (C, T, 0) | -6.823 | -3.4692 | 0 | 0.0017 | 0.9016 | -0.0821 | 0.892 |
|  |  | (C, 0, 0) | -6.8691 | -2.8996 | 0 |  |  | -0.0172 | 0.954 |
|  |  | (0, 0, 0) | -6.9144 | -1.9451 | 0 |  |  |  |  |
| PMI_3 | Raw | (C, T, 2) | -4.7487 | -3.4709 | 0.0013 | -0.0439 | 0.0023 | 37.143 | 0 |
|  |  | (C, 0, 2) | -3.3313 | -2.9007 | 0.0169 |  |  | 19.184 | 0.0014 |
|  |  | (0, 0, 9) | -1.3149 | -1.9457 | 0.1726 |  |  |  |  |
|  | Detrending | (C, T, 2) | -4.7487 | -3.4709 | 0.0013 | 0.0013 | 0.8956 | -0.0645 | 0.8888 |
|  |  | (C, 0, 2) | -4.7813 | -2.9007 | 0.0002 |  |  | -0.0113 | 0.9588 |
|  |  | (0, 0, 2) | -4.8151 | -1.9452 | 0 |  |  |  |  |
| RECI_1 | Raw | (C, T, 1) | -2.6566 | -3.47 | 0.2574 | -0.0068 | 0.4101 | 12.7861 | 0.0099 |
|  |  | (C, 0, 1) | -2.5459 | -2.9001 | 0.1089 |  |  | 11.7077 | 0.0137 |
|  |  | (0, 0, 1) | -0.5321 | -1.9451 | 0.4832 |  |  |  |  |
|  | Difference | (C, T, 0) | -6.0753 | -3.47 | 0 | -0.0023 | 0.7832 | 0.011 | 0.9768 |
|  |  | (C, 0, 0) | -6.1078 | -2.9001 | 0 |  |  | -0.0803 | 0.6624 |
|  |  | (0, 0, 0) | -6.1251 | -1.9451 | 0 |  |  |  |  |
| RECI_2 | Raw | (C, T, 1) | -2.5805 | -3.47 | 0.2902 | -0.0076 | 0.3962 | 13.3064 | 0.012 |
|  |  | (C, 0, 1) | -2.4569 | -2.9001 | 0.1301 |  |  | 12.048 | 0.0172 |
|  |  | (0, 0, 1) | -0.5135 | -1.9451 | 0.4909 |  |  |  |  |
|  | Difference | (C, T, 0) | -6.55 | -3.47 | 0 | -0.0025 | 0.7815 | 0.0159 | 0.9689 |
|  |  | (C, 0, 0) | -6.5854 | -2.9001 | 0 |  |  | -0.0828 | 0.6741 |
|  |  | (0, 0, 0) | -6.6082 | -1.9451 | 0 |  |  |  |  |
| RECI_3 | Raw | (C, T, 1) | -2.6902 | -3.47 | 0.2436 | -0.007 | 0.4217 | 13.4851 | 0.0089 |
|  |  | (C, 0, 1) | -2.5889 | -2.9001 | 0.0997 |  |  | 12.438 | 0.0121 |
|  |  | (0, 0, 1) | -0.4552 | -1.9451 | 0.5147 |  |  |  |  |
|  | Difference | (C, T, 0) | -6.2831 | -3.47 | 0 | -0.0025 | 0.7784 | 0.0293 | 0.9418 |
|  |  | (C, 0, 0) | -6.3163 | -2.9001 | 0 |  |  | -0.0696 | 0.7208 |
|  |  | (0, 0, 0) | -6.3434 | -1.9451 | 0 |  |  |  |  |
| R_1 | Raw | (C, T, 2) | -3.0557 | -3.4709 | 0.1246 | -0.0028 | 0.3183 | 0.7582 | 0.0041 |
|  |  | (C, 0, 2) | -2.9561 | -2.9007 | 0.0438 |  |  | 0.6215 | 0.0053 |
|  |  | (0, 0, 1) | -0.7186 | -1.9451 | 0.4022 |  |  |  |  |
| R_2 | Raw | (C, T, 0) | -3.3419 | -3.4692 | 0.0673 | -0.0008 | 0.726 | 0.619 | 0.0044 |
|  |  | (C, 0, 0) | -3.3458 | -2.8996 | 0.0161 |  |  | 0.5829 | 0.0021 |
|  |  | (0, 0, 0) | -0.9521 | -1.9451 | 0.3015 |  |  |  |  |
| R_3 | Raw | (C, T, 1) | -3.1362 | -3.47 | 0.1056 | -0.0029 | 0.4424 | 0.8971 | 0.0054 |
|  |  | (C, 0, 1) | -3.08 | -2.9001 | 0.0323 |  |  | 0.7631 | 0.0044 |
|  |  | (0, 0, 1) | -0.8715 | -1.9451 | 0.3353 |  |  |  |  |
| STOCK_1 | Raw | (C, T, 4) | -3.4592 | -3.4726 | 0.0516 | 1.4332 | 0.5511 | 865.2641 | 0.0006 |
|  |  | (C, 0, 4) | -3.5292 | -2.9018 | 0.0098 |  |  | 862.6164 | 0.0006 |
|  |  | (0, 0, 8) | 0.062 | -1.9456 | 0.6992 |  |  |  |  |
| STOCK_2 | Raw | (C, T, 2) | -5.0039 | -3.4709 | 0.0006 | 3.3299 | 0.1133 | 811.563 | 0 |
|  |  | (C, 0, 4) | -3.6386 | -2.9018 | 0.0072 |  |  | 817.7936 | 0.0004 |
|  |  | (0, 0, 8) | 0.0432 | -1.9456 | 0.6932 |  |  |  |  |
| STOCK_3 | Raw | (C, T, 5) | -3.4403 | -3.4734 | 0.0541 | 1.7218 | 0.4951 | 1015.063 | 0.0007 |
|  |  | (C, 0, 5) | -3.4922 | -2.9024 | 0.0109 |  |  | 997.5015 | 0.0007 |
|  |  | (0, 0, 8) | -0.0325 | -1.9456 | 0.6685 |  |  |  |  |
| GDP | Raw | (C, T, 3) | -6.4028 | -3.4717 | 0 | -0.12 | 0 | 13.1403 | 0 |
|  |  | (C, 0, 8) | -1.361 | -2.9042 | 0.5964 |  |  | 0.8227 | 0.4083 |
|  |  | (0, 0, 8) | -1.8832 | -1.9456 | 0.0573 |  |  |  |  |
|  | Detrending | (C, T, 3) | -6.4028 | -3.4717 | 0 | -0.0039 | 0.7531 | 0.1612 | 0.7739 |
|  |  | (C, 0, 3) | -6.4385 | -2.9012 | 0 |  |  | 0.005 | 0.9847 |
|  |  | (0, 0, 3) | -6.4855 | -1.9453 | 0 |  |  |  |  |
| UE | Raw | (C, T, 1) | -3.6896 | -3.47 | 0.0291 | -0.0013 | 0.0207 | 1.2125 | 0.0004 |
|  |  | (C, 0, 1) | -2.7581 | -2.9001 | 0.0692 |  |  | 0.7244 | 0.0078 |
|  |  | (0, 0, 4) | -0.8196 | -1.9453 | 0.3576 |  |  |  |  |
|  | Detrending | (C, T, 1) | -3.6896 | -3.47 | 0.0291 | -0.0001 | 0.8493 | 0.0021 | 0.9188 |
|  |  | (C, 0, 1) | -3.7189 | -2.9001 | 0.0056 |  |  | -0.0013 | 0.894 |
|  |  | (0, 0, 1) | -3.7456 | -1.9451 | 0.0003 |  |  |  |  |
| ECI_1 | Raw | (C, T, 3) | -4.2432 | -3.5005 | 0.0077 | 0.0032 | 0.727 | 36.6796 | 0.0001 |
|  |  | (C, 0, 3) | -4.2868 | -2.92 | 0.0013 |  |  | 36.8672 | 0.0001 |
|  |  | (0, 0, 5) | -0.0474 | -1.9477 | 0.6621 |  |  |  |  |
| ECI_2 | Raw | (C, T, 3) | -3.7177 | -3.5005 | 0.03 | -0.0001 | 0.9948 | 34.1572 | 0.0006 |
|  |  | (C, 0, 3) | -3.7594 | -2.92 | 0.0059 |  |  | 34.1526 | 0.0005 |
|  |  | (0, 0, 1) | -0.6725 | -1.9471 | 0.421 |  |  |  |  |
| ECI_3 | Raw | (C, T, 0) | -5.9672 | -3.4953 | 0 | 0.0034 | 0.726 | 33.2973 | 0 |
|  |  | (C, 0, 0) | -6.0979 | -2.9166 | 0 |  |  | 33.6957 | 0 |
|  |  | (0, 0, 1) | -0.5166 | -1.9471 | 0.4881 |  |  |  |  |

Note: (1) Because Mixed-frequency Granger casualty test and RU-MIDAS use CCI_1, CC_2 and CCI_3 which indicate the first, second and third month CCI within a quarter, we adopt CCI_1, CCI_2 and CCI_3 rather than CCI in ADF test. (2) Column Category indicates the type of value – raw, first-order differenced and subtracted the deterministic trend from the raw value. (3) Column Form suggests the test form. (C, T, L) indicates the regression contains the constant term, trend term and lags. The optimal lag order is selected by AIC. (4) The column Statistics indicates the $\boldsymbol{\tau}$-statistics (Dickey & Fuller, 1979). Column CV5 indicates the critical value at 5%. (4) Column Trend, Trend_p, Constant, and Constant_p report the coefficient and responding p-value of the trend and constant in the test model.
