# Supplementary material for: Confidence, animal spirits, and the macroeconomy in China: Based on mixed-frequency data models
Source: PLoS One. 2025 Sep 19;20(9):e0332909. doi: 10.1371/journal.pone.0332909 (PMC12448974; doi:10.1371/journal.pone.0332909)
Supplement: S2 Table — (DOCX) [file pone.0332909.s002.docx]

**S2 Table. Correlation matrix.**

|  | CCI_1 | CCI_2 | CCI_3 | CPI_1 | CPI_2 | CPI_3 | PMI_1 | PMI_2 | PMI_3 | RECI_1 | RECI_2 | RECI_3 | R_1 | R_2 | R_3 | Stock_1 | Stock_2 | Stock_3 | GDP | UE |
| --- | --- | --- | --- | --- | --- | --- | --- | --- | --- | --- | --- | --- | --- | --- | --- | --- | --- | --- | --- | --- |
| CCI_1 | 1 | 0.7368 | 0.2862 | -0.1438 | -0.1135 | -0.0800 | 0.1798 | 0.2180 | 0.2595 | 0.1707 | 0.1405 | 0.1394 | 0.0822 | 0.1469 | 0.0259 | 0.1056 | 0.1193 | 0.1415 | 0.2509 | -0.0711 |
| CCI_2 | 0.8642 | 1 | 0.4531 | -0.1436 | -0.1629 | -0.1275 | 0.2312 | 0.2764 | 0.2271 | 0.2602 | 0.3344 | 0.2486 | 0.1635 | 0.2101 | 0.1531 | 0.0513 | 0.0633 | 0.1056 | 0.1998 | 0.0068 |
| CCI_3 | 0.6651 | 0.6871 | 1 | -0.1755 | -0.1773 | -0.1677 | 0.2275 | 0.2548 | 0.1826 | 0.4008 | 0.4524 | 0.4251 | 0.1484 | 0.1706 | 0.0864 | 0.0223 | 0.0280 | 0.0827 | 0.2483 | -0.0414 |
| CPI_1 | -0.1088 | -0.1191 | -0.1518 | 1 | 0.8859 | 0.8427 | -0.1471 | -0.1155 | -0.1640 | -0.1635 | -0.2058 | -0.2299 | 0.4240 | 0.3698 | 0.3862 | 0.1277 | 0.1395 | -0.0119 | -0.2477 | -0.2465 |
| CPI_2 | -0.0680 | -0.1217 | -0.1264 | 0.9184 | 1 | 0.9219 | -0.0557 | -0.0612 | -0.0603 | -0.0643 | -0.1387 | -0.1733 | 0.3794 | 0.3457 | 0.4181 | 0.2239 | 0.2043 | 0.0637 | -0.0964 | -0.3179 |
| CPI_3 | -0.0572 | -0.0976 | -0.1214 | 0.8870 | 0.9639 | 1 | -0.0542 | -0.0217 | -0.0435 | -0.0761 | -0.1461 | -0.1777 | 0.4036 | 0.3750 | 0.4490 | 0.1925 | 0.1989 | 0.0921 | -0.0377 | -0.3436 |
| PMI_1 | 0.1781 | 0.1898 | 0.2246 | -0.0436 | 0.1202 | 0.1650 | 1 | 0.7602 | 0.4426 | 0.2882 | 0.3484 | 0.2799 | -0.2010 | -0.1472 | -0.1074 | 0.3259 | 0.3160 | 0.2910 | 0.5804 | -0.1044 |
| PMI_2 | 0.0900 | 0.2485 | 0.2281 | -0.2364 | -0.1032 | -0.0048 | 0.6005 | 1 | 0.6655 | 0.3597 | 0.4368 | 0.3838 | -0.1993 | -0.1474 | -0.0627 | 0.3918 | 0.3938 | 0.3812 | 0.6417 | -0.0833 |
| PMI_3 | 0.0788 | 0.1260 | 0.1591 | -0.1110 | 0.0503 | 0.1169 | 0.4843 | 0.5850 | 1 | 0.2789 | 0.2610 | 0.2738 | -0.2931 | -0.2868 | -0.2628 | 0.3162 | 0.3496 | 0.3649 | 0.5938 | -0.1619 |
| RECI_1 | 0.1687 | 0.1910 | 0.3395 | -0.1794 | -0.0849 | -0.0548 | 0.3401 | 0.3340 | 0.3275 | 1 | 0.8218 | 0.6793 | -0.0798 | -0.0311 | 0.0304 | 0.2894 | 0.2249 | 0.2441 | 0.4534 | -0.0075 |
| RECI_2 | 0.1367 | 0.2336 | 0.3407 | -0.2717 | -0.2026 | -0.1629 | 0.3450 | 0.4740 | 0.2923 | 0.8911 | 1 | 0.8832 | -0.1521 | -0.1122 | -0.0371 | 0.1971 | 0.1415 | 0.1905 | 0.3523 | 0.0079 |
| RECI_3 | 0.1235 | 0.1990 | 0.3381 | -0.3017 | -0.2619 | -0.2183 | 0.3067 | 0.4610 | 0.3466 | 0.8341 | 0.9453 | 1 | -0.1265 | -0.1590 | -0.0998 | 0.2234 | 0.1938 | 0.2424 | 0.3055 | -0.0068 |
| R_1 | 0.1436 | 0.1500 | 0.1743 | 0.4536 | 0.4141 | 0.4272 | -0.0667 | -0.0963 | -0.2118 | -0.1454 | -0.1941 | -0.1759 | 1 | 0.8824 | 0.7807 | -0.0676 | -0.0738 | -0.1203 | -0.0998 | -0.3305 |
| R_2 | 0.2050 | 0.1792 | 0.1996 | 0.4025 | 0.3963 | 0.4096 | -0.0384 | -0.0526 | -0.2038 | -0.0312 | -0.1047 | -0.1498 | 0.8499 | 1 | 0.8482 | -0.1421 | -0.1461 | -0.1703 | -0.0810 | -0.3119 |
| R_3 | 0.1073 | 0.0589 | 0.0460 | 0.3394 | 0.3627 | 0.3957 | 0.0153 | 0.0816 | -0.1420 | 0.0582 | 0.0025 | -0.0460 | 0.6278 | 0.7864 | 1 | -0.0848 | -0.1113 | -0.1431 | -0.0193 | -0.2787 |
| Stock_1 | 0.0259 | 0.0044 | -0.0006 | 0.3129 | 0.4345 | 0.4374 | 0.2334 | 0.2531 | 0.3223 | 0.2048 | 0.1897 | 0.1785 | -0.0114 | -0.0629 | -0.0415 | 1 | 0.9585 | 0.8714 | 0.3665 | -0.1289 |
| Stock_2 | 0.0094 | 0.0069 | -0.0180 | 0.2790 | 0.3935 | 0.4113 | 0.2393 | 0.2610 | 0.3450 | 0.1748 | 0.1563 | 0.1632 | -0.0216 | -0.0798 | -0.0383 | 0.9585 | 1 | 0.9311 | 0.3505 | -0.0971 |
| Stock_3 | -0.0037 | 0.0076 | -0.0007 | 0.1570 | 0.2815 | 0.3145 | 0.2263 | 0.2869 | 0.3418 | 0.1852 | 0.2058 | 0.2159 | -0.0721 | -0.1121 | -0.0705 | 0.9151 | 0.9633 | 1 | 0.3360 | -0.0321 |
| GDP | 0.2280 | 0.2936 | 0.2723 | -0.2237 | -0.1042 | -0.0262 | 0.3979 | 0.6187 | 0.3027 | 0.2766 | 0.3430 | 0.2842 | -0.0093 | 0.0185 | 0.0562 | 0.2643 | 0.2673 | 0.2830 | 1 | -0.3210 |
| UE | -0.1588 | -0.0951 | -0.1172 | -0.3605 | -0.4173 | -0.3963 | -0.0557 | 0.1342 | -0.0742 | -0.0396 | -0.0005 | 0.0028 | -0.2382 | -0.2829 | -0.1866 | -0.1219 | -0.0907 | -0.0805 | -0.0152 | 1 |

Note: The lower triangle of the correlation matrix reports the Pearson coefficients, and the upper triangle reports the Spearman coefficients of variables.
