# Supplementary material for: Confidence, animal spirits, and the macroeconomy in China: Based on mixed-frequency data models
Source: PLoS One. 2025 Sep 19;20(9):e0332909. doi: 10.1371/journal.pone.0332909 (PMC12448974; doi:10.1371/journal.pone.0332909)
Supplement: S3 Table — (DOCX) [file pone.0332909.s003.docx]

## S3 Table

Estimation results of RU-MIDAS (Economist Confidence Index)

|  | ECI_*i*  (*i*=1) | ECI_*i*  (*i*=2) | ECI*_i*  (*i*=3) |
| --- | --- | --- | --- |
| L(ECI_i, 1) | -0.0406 | 0.0734 | -0.4839^***^ |
|  | (0.1133) | (0.1287) | (0.1295) |
| L(ECI_i, 2) | 0.3042^**^ |  | -0.1456 |
|  | (0.1360) |  | (0.1199) |
| L(ECI_i, 3) |  |  | -0.1782^*^ |
|  |  |  | (0.1028) |
| CPI_i | 0.0127 | -0.1661 | 0.1271 |
|  | (0.1050) | (0.1290) | (0.1954) |
| L(CPI_i, 1) |  | 0.1983 | -0.1817 |
|  |  | (0.1498) | (0.2443) |
| L(CPI_i, 2) |  | -0.1964 | 0.0796 |
|  |  | (0.1637) | (0.2345) |
| L(CPI_i, 3) |  | 0.4799^***^ | 0.2201 |
|  |  | (0.1351) | (0.1730) |
| PMI_i | 0.3812^***^ | 0.0132 | 0.2483^**^ |
|  | (0.0937) | (0.0596) | (0.0963) |
| L(PMI_i, 1) |  | -0.0829 | 0.1025 |
|  |  | (0.0595) | (0.0911) |
| L(PMI_i, 2) |  | -0.1622^**^ | 0.1470 |
|  |  | (0.0659) | (0.0988) |
| L(PMI_i, 3) |  |  | 0.3156^***^ |
|  |  |  | (0.0972) |
| RECI_i | 0.0941 | 0.0034 | 0.1771^***^ |
|  | (0.0567) | (0.0660) | (0.0591) |
| L(RECI_i, 1) |  | 0.1816^**^ |  |
|  |  | (0.0694) |  |
| L(RECI_i, 2) |  | 0.0908 |  |
|  |  | (0.0631) |  |
| L(RECI_i, 3) |  | 0.1009 |  |
|  |  | (0.0608) |  |
| R_i | -0.1078 | -0.3277 | 0.1570 |
|  | (0.1690) | (0.2143) | (0.1387) |
| L(R_i, 3) |  |  | 0.2714^*^ |
|  |  |  | (0.1329) |
| Stock_i | -0.0002 | -0.0002 | -0.0001 |
|  | (0.0003) | (0.0003) | (0.0003) |
| L(Stock_i, 1) | 0.0001 |  | -0.0003 |
|  | (0.0004) |  | (0.0004) |
| L(Stock_i, 2) | -0.0006^*^ |  | -0.0005 |
|  | (0.0003) |  | (0.0003) |
| GDP | 0.0524 | 0.1372^**^ | 0.2435^***^ |
|  | (0.0490) | (0.0521) | (0.0493) |
| L(GDP, 1) | 0.1583^***^ | 0.0877 | 0.1042^*^ |
|  | (0.0398) | (0.0579) | (0.0528) |
| L(GDP, 2) | -0.0115 | 0.1254^**^ | 0.0492 |
|  | (0.0464) | (0.0519) | (0.0477) |
| L(GDP, 3) | -0.1237^***^ | -0.0830^*^ | -0.0664 |
|  | (0.0407) | (0.0473) | (0.0441) |
| UE | 1.5844^**^ | 4.0889^***^ | 4.5131^***^ |
|  | (0.6974) | (1.3710) | (1.0705) |
| L(UE, 1) |  | -4.2603^**^ | 0.1231 |
|  |  | (1.9345) | (1.4780) |
| L(UE, 2) |  | 1.6685 | -3.3238^**^ |
|  |  | (1.2786) | (1.4926) |
| L(UE, 3) |  |  | 4.9491^***^ |
|  |  |  | (1.2994) |
| Constant | 39.3419^***^ | 48.1334^***^ | 93.1400^***^ |
|  | (8.6973) | (6.5993) | (12.8125) |
| Observations | 52 | 52 | 52 |
| Adjusted R^2^ | 0.6696 | 0.6984 | 0.7619 |
| F Statistic | 8.3841^***^   (df = 14; 37) | 6.6229^***^   (df = 21; 30) | 7.5294^***^  (df = 25; 26) |

Note: (1) This table reports the estimation results of RU-MIDAS with ECI as the dependent variable for $i=1, 2, 3$ in equation (6). L(CCI_i, 1) and L(GDP, 1) represent the lagged CCI_i and UE (i = 1, 2, and 3), respectively. The same applies to the other variables. (2) t-statistic in parentheses and p-value in brackets. * p < 0.1, ** p < 0.05, ***p < 0.01.
