# Supplementary material for: Confidence, animal spirits, and the macroeconomy in China: Based on mixed-frequency data models
Source: PLoS One. 2025 Sep 19;20(9):e0332909. doi: 10.1371/journal.pone.0332909 (PMC12448974; doi:10.1371/journal.pone.0332909)
Supplement: S4 Table — (DOCX) [file pone.0332909.s004.docx]

**S4 Tables.**

Results of RU-MIDAS with subsamples.

|  | CCI_*i*  ($i=1$) | | | CCI_*i*  ($i=2$) | | | CCI_*i*  ($i=3$) | | |
| --- | --- | --- | --- | --- | --- | --- | --- | --- | --- |
|  | (1)  Subsample1 | (2)  Subsample2 | (3)  Subsample3 | (4)  Subsample1 | (5)  Subsample2 | (6)  Subsample3 | (7)  Subsample1 | (8)  Subsample2 | (9)  Subsample3 |
| L(CCI_i, 1) | -0.3182^*^ | -0.3037^*^ | -0.2687^*^ | -0.2372 | -0.1824 | -0.1824 | -0.1840 | -0.3792^**^ | -0.3566^*^ |
|  | (0.1643) | (0.1763) | (0.1522) | (0.1565) | (0.1375) | (0.1375) | (0.1854) | (0.1805) | (0.1822) |
| L(CCI_i, 2) | -0.2933^*^ | -0.2924 |  | -0.3964^**^ | -0.4660^***^ | -0.4660^***^ | -0.1682 | -0.4032^**^ | -0.3982^**^ |
|  | (0.1711) | (0.1752) |  | (0.1610) | (0.1455) | (0.1455) | (0.1734) | (0.1793) | (0.1796) |
| L(CCI_i, 3) | -0.1865 |  |  | 0.0514 | 0.1687 | 0.1687 | -0.3089^*^ | -0.3058^*^ | -0.3020^*^ |
|  | (0.1691) |  |  | (0.1632) | (0.1455) | (0.1455) | (0.1638) | (0.1506) | (0.1508) |
| CPI_i | -0.8490^*^ | 0.0531 | 0.0609 | -0.9052^*^ | -0.6649 | -0.6649 | 1.0011 | 0.4596 | 0.4944 |
|  | (0.4319) | (0.7375) | (0.6191) | (0.4764) | (0.3966) | (0.3966) | (0.6894) | (0.7068) | (0.7086) |
| L(CPI_i, 1) |  | -0.7803 | -1.3749^*^ |  |  |  | -0.8924 | 0.9975 | 0.4818 |
|  |  | (0.9978) | (0.7858) |  |  |  | (0.9298) | (0.7178) | (0.8922) |
| L(CPI_i, 2) |  | 0.4652 | 0.5324 |  |  |  | -0.5295 |  | 0.6379 |
|  |  | (0.7750) | (0.8150) |  |  |  | (0.8347) |  | (0.6540) |
| L(CPI_i, 3) |  |  | 0.8303 |  |  |  | 1.1413 |  |  |
|  |  |  | (0.5739) |  |  |  | (0.7147) |  |  |
| PMI_i | -0.1011 | -0.0505 | -0.2810 | 0.5539 | 0.3125 | 0.3125 | -0.5530 | 0.1191 | 0.0096 |
|  | (0.3081) | (0.4547) | (0.3068) | (0.4859) | (0.4220) | (0.4220) | (0.4981) | (0.3662) | (0.3835) |
| L(PMI_i, 1) |  | -0.2543 |  |  | 0.3300 | 0.3300 | 0.2850 | 0.5105 | 0.6476 |
|  |  | (0.4040) |  |  | (0.3671) | (0.3671) | (0.3522) | (0.3573) | (0.3844) |
| L(PMI_i, 2) |  | 0.4554 |  |  | 0.8884^**^ | 0.8884^**^ | 0.5367 | 0.6657^*^ | 0.6168 |
|  |  | (0.4514) |  |  | (0.3723) | (0.3723) | (0.3871) | (0.3550) | (0.3590) |
|  |  |  |  |  |  |  |  | 0.7032 | 0.6119 |
|  |  |  |  |  |  |  |  | (0.4168) | (0.4277) |
| RECI_i | -0.0299 | 0.0508 | -0.1886 | 0.1911 | 0.3532 | 0.3532 | 0.9281^***^ | 0.7433^**^ | 0.7340^**^ |
|  | (0.3102) | (0.3489) | (0.2864) | (0.3163) | (0.2811) | (0.2811) | (0.2835) | (0.2605) | (0.2610) |
| L(RECI_i, 1) | 0.4710 | 0.5172 | 0.5263^*^ | 0.2350 |  |  | 0.5264^*^ | 0.8921^***^ | 0.8080^**^ |
|  | (0.2990) | (0.3264) | (0.2791) | (0.3019) |  |  | (0.2855) | (0.2860) | (0.2990) |
| L(RECI_i, 2) | -0.0505 | 0.0769 | -0.0077 |  |  |  | 0.6762^**^ | 1.0455^***^ | 1.1262^***^ |
|  | (0.2711) | (0.3321) | (0.2743) |  |  |  | (0.2818) | (0.2716) | (0.2842) |
| L(RECI_i, 3) | 0.7838^***^ | 0.8397^***^ | 0.7408^***^ |  |  |  | 0.5557^*^ | 0.6283^**^ | 0.6214^**^ |
|  | (0.2740) | (0.2909) | (0.2584) |  |  |  | (0.2896) | (0.2408) | (0.2412) |
| R_i | 0.9402 | -0.3269 | 0.1046 | 0.4549 | 0.8250 | 0.8250 | -1.4299^**^ | -1.7191^***^ | -1.7523^***^ |
|  | (0.9094) | (1.2319) | (0.8409) | (1.2286) | (0.8963) | (0.8963) | (0.5923) | (0.5436) | (0.5454) |
| L(R_i, 1) |  | 0.6382 |  | 1.8667^*^ |  |  | -0.0927 | -1.0005 | -0.9223 |
|  |  | (0.9877) |  | (1.0424) |  |  | (0.6845) | (0.7458) | (0.7511) |
| L(R_i, 2) |  | 0.6279 |  |  |  |  | 0.6554 | 0.0346 | -0.0784 |
|  |  | (1.1210) |  |  |  |  | (0.6702) | (0.6739) | (0.6846) |
|  |  |  |  |  |  |  | 0.8942 | 1.6045^**^ | 1.5032^**^ |
|  |  |  |  |  |  |  | (0.5760) | (0.5855) | (0.5954) |
| Stock_i | 0.0007 | 0.0003 | 0.0003 | 0.0011 | 0.0034^***^ | 0.0034^***^ | 0.0009 | 0.0007 | 0.0006 |
|  | (0.0007) | (0.0008) | (0.0006) | (0.0009) | (0.0011) | (0.0011) | (0.0011) | (0.0010) | (0.0010) |
|  |  |  |  |  | -0.0028^**^ | -0.0028^**^ | -0.0003 | 0.0016 | 0.0017 |
|  |  |  |  |  | (0.0011) | (0.0011) | (0.0015) | (0.0014) | (0.0014) |
|  |  |  |  |  |  |  | -0.0017 | -0.0015 | -0.0013 |
|  |  |  |  |  |  |  | (0.0012) | (0.0012) | (0.0012) |
|  |  |  |  |  |  |  |  | -0.0028^**^ | -0.0030^**^ |
|  |  |  |  |  |  |  |  | (0.0011) | (0.0011) |
| GDP | -0.1161 | -0.2465 | 0.5062 | -0.3770 | -1.6172^**^ | -1.6172^**^ | -0.4343 | -0.6862 | -0.4714 |
|  | (0.5423) | (1.4944) | (0.6061) | (0.6213) | (0.7545) | (0.7545) | (0.9705) | (0.9555) | (0.9817) |
| L(GDP, 1) |  | 1.1570 |  |  |  |  | -0.2236 | -2.1887 | -1.9476 |
|  |  | (1.8164) |  |  |  |  | (1.3791) | (1.3810) | (1.4046) |
| L(GDP, 2) |  | -1.4902 |  |  |  |  |  | -1.5012 | -1.8019 |
|  |  | (1.4380) |  |  |  |  |  | (1.0377) | (1.0838) |
| UE | -11.2642 | -21.0598 | -10.9188 | -12.7769 | -4.4784 | -4.4784 | -8.6242 | -20.2631 | -22.6268^*^ |
|  | (11.5425) | (15.4524) | (11.6928) | (13.1304) | (4.2929) | (4.2929) | (12.2624) | (11.9636) | (12.2217) |
| L(UE, 1) | -4.4942 | 18.5604 | -14.8947 | 4.4475 |  |  | 8.0957 | 23.4590 | 23.4120 |
|  | (15.5711) | (17.2624) | (15.4709) | (15.8193) |  |  | (13.2642) | (14.2600) | (14.2784) |
| L(UE, 2) | 25.7421 |  | 30.3795^**^ | 12.6172 |  |  |  | 10.3890 | 11.1433 |
|  | (15.2522) |  | (12.3857) | (14.9778) |  |  |  | (12.8453) | (12.8850) |
| L(UE, 3) | -15.6863 |  |  | -10.3026 |  |  |  | -22.2372^*^ | -19.5288 |
|  | (14.8237) |  |  | (13.9887) |  |  |  | (12.4218) | (12.7440) |
| Constant | -3.7306 | -2.6475 | -0.7234 | -8.3439^*^ | -3.6038 | -3.6038 | 3.6395 | 9.0520^**^ | 9.3144^**^ |
|  | (3.4851) | (3.8168) | (3.1485) | (4.2483) | (3.6724) | (3.6724) | (3.5294) | (4.2657) | (4.2797) |
| Observations | 48 | 48 | 48 | 48 | 48 | 48 | 48 | 48 | 48 |
| Adjusted R^2^ | 0.1527 | 0.0603 | 0.1973 | 0.2035 | 0.3225 | 0.3225 | 0.2784 | 0.4323 | 0.4308 |
| F Statistic | 1.5292  (df = 16; 31) | 1.1437  (df = 21; 26) | 1.7219^*^  (df = 16; 31) | 1.8007^*^  (df = 15; 32) | 2.7208^***^  (df = 13; 34) | 2.7208^***^  (df = 13; 34) | 1.7253  (df = 25; 22) | 2.2781^**^  (df = 28; 19) | 2.2267^**^  (df = 29; 18) |

Note: (1) This table reports the estimation results of RU-MIDAS for $i=1, 2, 3$ in equation (6). L(CCI_*i*, 1) and L(GDP, 1) represent the lagged CCI_*i* and UE, respectively. The same applies to the other variables. See Table 1 for definitions of variables. (2) *t-*statistic in parentheses and *p-*value in brackets. * p < 0.1, ** p < 0.05, ***p < 0.01. (3) Subsample1, Subsample2 and Subsample3 indicate the subsamples including the global finance crisis but excluding COVID-19, including COVID-19 but excluding the global finance crisis, and excluding both the global finance crisis and COVID-19, respectively. The global financial crisis spanned from September 2008 to July 2010. COVID-19 spans from January 2020 to June 2024.
